# 2-Chloro-*N*′-(4-nitro­benzyl­idene)benzo­hydrazide

**DOI:** 10.1107/S1600536809055330

**Published:** 2010-01-09

**Authors:** Cong-Shan Zhou, Tao Yang

**Affiliations:** a College of Chemistry and Chemical Engineering, Hunan Institute of Science and Technology, Yueyang, Hunan 414006, People’s Republic of China

## Abstract

The title Schiff base compound, C_14_H_10_ClN_3_O_3_, exists in a *trans* configuration with respect to the C=N bond. The dihedral angle between the two benzene rings is 15.9 (2)°. In the crystal, the mol­ecules are linked into chains along [101] by inter­molecular N—H⋯O hydrogen bonds.

## Related literature

For the biological properties of Schiff bases, see: Mohamed *et al.* (2009 ▶); Ritter *et al.* (2009 ▶); Bagihalli *et al.* (2008 ▶). For the crystal structures of Schiff base compounds, see: Fun *et al.* (2008 ▶); Shafiq *et al.* (2009 ▶); Goh *et al.* (2010 ▶). For other related structures, see: Zhou *et al.* (2009 ▶); Zhou & Yang (2009 ▶).
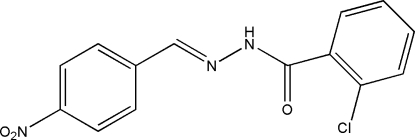


## Experimental

### 

#### Crystal data

C_14_H_10_ClN_3_O_3_
*M* *_r_* = 303.70
Monoclinic, 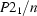

*a* = 7.2752 (3) Å
*b* = 26.4081 (9) Å
*c* = 7.7284 (3) Å
β = 113.000 (2)°
*V* = 1366.78 (9) Å^3^
*Z* = 4
Mo *K*α radiation
μ = 0.29 mm^−1^
*T* = 298 K
0.23 × 0.20 × 0.20 mm

#### Data collection

Bruker SMART 1000 CCD area-detector diffractometer
Absorption correction: multi-scan (*SADABS*; Sheldrick, 1996 ▶) *T*_min_ = 0.936, *T*_max_ = 0.944
7876 measured reflections
2763 independent reflections
1934 reflections with *I* > 2σ(*I*)
*R*_int_ = 0.036

#### Refinement

*R*[*F* ^2^ > 2σ(*F* ^2^)] = 0.044
*wR*(*F* ^2^) = 0.113
*S* = 1.03
2763 reflections
193 parameters
1 restraint
H atoms treated by a mixture of independent and constrained refinement
Δρ_max_ = 0.19 e Å^−3^
Δρ_min_ = −0.27 e Å^−3^

### 

Data collection: *SMART* (Bruker, 2007 ▶); cell refinement: *SAINT* (Bruker, 2007 ▶); data reduction: *SAINT*; program(s) used to solve structure: *SHELXTL* (Sheldrick, 2008 ▶); program(s) used to refine structure: *SHELXTL*; molecular graphics: *SHELXTL*; software used to prepare material for publication: *SHELXTL*.

## Supplementary Material

Crystal structure: contains datablocks global, I. DOI: 10.1107/S1600536809055330/ci5003sup1.cif

Structure factors: contains datablocks I. DOI: 10.1107/S1600536809055330/ci5003Isup2.hkl

Additional supplementary materials:  crystallographic information; 3D view; checkCIF report

## Figures and Tables

**Table 1:** Hydrogen-bond geometry (Å, °)

*D*—H⋯*A*	*D*—H	H⋯*A*	*D*⋯*A*	*D*—H⋯*A*
N2—H2*A*⋯O3^i^	0.90 (1)	1.97 (1)	2.855 (2)	169 (2)

Symmetry code: (i) 
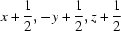
.

**Fig. 1.**
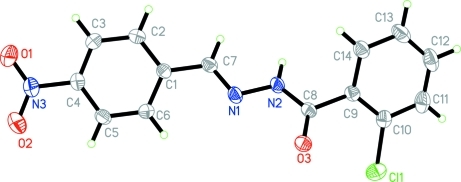
The molecular structure of the title compound, with displacement ellipsoids drawn at the 30% probability level.

**Fig. 2.**
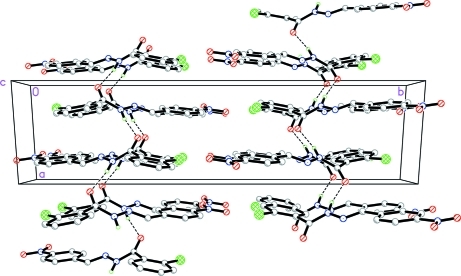
The molecular packing of the title compound, viewed along the c axis. Hydrogen bonds are shown as dashed lines.

## supplementary crystallographic information

### Comment

Schiff bases are a kind of interesting compounds, which possess excellent biological properties, such as antibacterial, antimicrobial, and antitumor (Mohamed *et al.*, 2009; Ritter *et al.*, 2009; Bagihalli *et al.*, 2008). Recently, a large number of Schiff bases derived from the reaction of aldehydes with benzohydrazides have been reported (Fun *et al.*, 2008; Shafiq *et al.*, 2009; Goh *et al.*, 2010). In this paper, the crystal structure of the title new Schiff base compound is reported.

In the title compound (Fig. 1), bond lengths are comparable with those observed in related structures (Zhou *et al.*, 2009; Zhou & Yang, 2009). The molecule exists in a *trans* configuration with respect to the acyclic C═N bond. The molecule is distorted from planarity, with a dihedral angle between the two benzene rings of 15.9 (2)°.

In the crystal structure, intermolecular N—H···O hydrogen bonds link adjacent molecules into chains along the [101] (Table 1 and Fig. 2).

### Experimental

4-Nitrobenzaldehyde (1.0 mmol, 151.0 mg) and 2-chlorobenzohydrazide (1.0 mmol, 170.0 mg) were dissolved in methanol (30 ml). The mixture was stirred for 30 min at room temperature. The resulting solution was left in air for a few days, yielding colourless block-shaped crystals.

### Refinement

Atom H2A was located in a difference map and refined with a N–H distance restraint of 0.90 (1) Å and U_iso_(H) = 0.08 Å^2^. The remaining H atoms were positioned geometrically (C–H = 0.93 Å) and refined using a riding model, with *U*_iso_(H) = 1.2*U*_eq_(C).

### Crystal data

**Table:**

C_14_H_10_ClN_3_O_3_	*F*(000) = 624
*M**_r_* = 303.70	*D*_x_ = 1.476 Mg m^−^^3^
Monoclinic, *P*2_1_/*n*	Mo *K*α radiation, λ = 0.71073 Å
Hall symbol: -P 2yn	Cell parameters from 1518 reflections
*a* = 7.2752 (3) Å	θ = 2.4–24.5°
*b* = 26.4081 (9) Å	µ = 0.29 mm^−^^1^
*c* = 7.7284 (3) Å	*T* = 298 K
β = 113.000 (2)°	Block, colourless
*V* = 1366.78 (9) Å^3^	0.23 × 0.20 × 0.20 mm
*Z* = 4	

### Data collection

**Table:**

Bruker SMART 1000 CCD area-detector diffractometer	2763 independent reflections
Radiation source: fine-focus sealed tube	1934 reflections with *I* > 2σ(*I*)
graphite	*R*_int_ = 0.036
ω scans	θ_max_ = 26.3°, θ_min_ = 1.5°
Absorption correction: multi-scan (*SADABS*; Sheldrick, 1996)	*h* = −9→9
*T*_min_ = 0.936, *T*_max_ = 0.944	*k* = −29→32
7876 measured reflections	*l* = −9→9

### Refinement

**Table:**

Refinement on *F*^2^	Primary atom site location: structure-invariant direct methods
Least-squares matrix: full	Secondary atom site location: difference Fourier map
*R*[*F*^2^ > 2σ(*F*^2^)] = 0.044	Hydrogen site location: inferred from neighbouring sites
*wR*(*F*^2^) = 0.113	H atoms treated by a mixture of independent and constrained refinement
*S* = 1.03	*w* = 1/[σ^2^(*F*_o_^2^) + (0.0488*P*)^2^ + 0.233*P*] where *P* = (*F*_o_^2^ + 2*F*_c_^2^)/3
2763 reflections	(Δ/σ)_max_ = 0.003
193 parameters	Δρ_max_ = 0.19 e Å^−^^3^
1 restraint	Δρ_min_ = −0.27 e Å^−^^3^

### Special details

**Table:**

Geometry. All esds (except the esd in the dihedral angle between two l.s. planes) are estimated using the full covariance matrix. The cell esds are taken into account individually in the estimation of esds in distances, angles and torsion angles; correlations between esds in cell parameters are only used when they are defined by crystal symmetry. An approximate (isotropic) treatment of cell esds is used for estimating esds involving l.s. planes.
Refinement. Refinement of F^2^ against ALL reflections. The weighted R-factor wR and goodness of fit S are based on F^2^, conventional R-factors R are based on F, with F set to zero for negative F^2^. The threshold expression of F^2^ > 2sigma(F^2^) is used only for calculating R-factors(gt) etc. and is not relevant to the choice of reflections for refinement. R-factors based on F^2^ are statistically about twice as large as those based on F, and R- factors based on ALL data will be even larger.

### Fractional atomic coordinates and isotropic or equivalent isotropic displacement parameters (Å^2^)

**Table:**

	*x*	*y*	*z*	*U*_iso_*/*U*_eq_	
Cl1	0.25382 (9)	0.09582 (2)	0.73742 (9)	0.0617 (2)	
N1	0.2283 (2)	0.28721 (7)	0.5887 (2)	0.0434 (4)	
N2	0.2675 (2)	0.25629 (7)	0.7434 (2)	0.0437 (4)	
N3	0.2493 (3)	0.47567 (8)	0.0679 (3)	0.0637 (6)	
O1	0.2747 (4)	0.52006 (8)	0.1124 (3)	0.1069 (8)	
O2	0.2156 (4)	0.46052 (8)	−0.0899 (3)	0.0983 (7)	
O3	0.0453 (2)	0.19654 (5)	0.57474 (19)	0.0498 (4)	
C1	0.2747 (3)	0.36895 (8)	0.4821 (3)	0.0388 (5)	
C2	0.3001 (3)	0.42028 (8)	0.5248 (3)	0.0505 (6)	
H2	0.3247	0.4311	0.6464	0.061*	
C3	0.2892 (3)	0.45526 (8)	0.3895 (3)	0.0525 (6)	
H3	0.3032	0.4896	0.4176	0.063*	
C4	0.2573 (3)	0.43846 (8)	0.2119 (3)	0.0447 (5)	
C5	0.2344 (3)	0.38808 (8)	0.1647 (3)	0.0469 (5)	
H5	0.2143	0.3776	0.0438	0.056*	
C6	0.2420 (3)	0.35350 (8)	0.3006 (3)	0.0443 (5)	
H6	0.2249	0.3193	0.2704	0.053*	
C7	0.2910 (3)	0.33266 (8)	0.6288 (3)	0.0435 (5)	
H7	0.3480	0.3427	0.7541	0.052*	
C8	0.1706 (3)	0.21195 (8)	0.7239 (3)	0.0396 (5)	
C9	0.2232 (3)	0.18436 (8)	0.9059 (3)	0.0392 (5)	
C10	0.2608 (3)	0.13269 (8)	0.9245 (3)	0.0452 (5)	
C11	0.3086 (3)	0.10918 (10)	1.0973 (4)	0.0612 (7)	
H11	0.3399	0.0749	1.1108	0.073*	
C12	0.3095 (4)	0.13687 (12)	1.2487 (4)	0.0680 (8)	
H12	0.3372	0.1207	1.3631	0.082*	
C13	0.2705 (4)	0.18759 (11)	1.2333 (3)	0.0623 (7)	
H13	0.2722	0.2058	1.3369	0.075*	
C14	0.2289 (3)	0.21163 (9)	1.0648 (3)	0.0496 (6)	
H14	0.2042	0.2463	1.0553	0.060*	
H2A	0.359 (3)	0.2671 (9)	0.853 (2)	0.080*	

### Atomic displacement parameters (Å^2^)

**Table:**

	*U*^11^	*U*^22^	*U*^33^	*U*^12^	*U*^13^	*U*^23^
Cl1	0.0603 (4)	0.0542 (4)	0.0718 (4)	0.0013 (3)	0.0270 (3)	−0.0108 (3)
N1	0.0437 (10)	0.0494 (11)	0.0319 (9)	−0.0017 (8)	0.0091 (7)	0.0068 (8)
N2	0.0462 (10)	0.0469 (10)	0.0281 (9)	−0.0076 (8)	0.0039 (7)	0.0049 (8)
N3	0.0825 (15)	0.0591 (14)	0.0531 (13)	0.0030 (11)	0.0302 (11)	0.0129 (11)
O1	0.197 (3)	0.0488 (12)	0.0902 (16)	−0.0089 (14)	0.0720 (17)	0.0117 (11)
O2	0.160 (2)	0.0860 (15)	0.0546 (12)	−0.0099 (14)	0.0489 (13)	0.0119 (11)
O3	0.0535 (9)	0.0502 (9)	0.0318 (8)	−0.0070 (7)	0.0017 (7)	0.0008 (7)
C1	0.0362 (10)	0.0422 (12)	0.0357 (11)	0.0003 (9)	0.0117 (9)	0.0005 (9)
C2	0.0632 (14)	0.0485 (13)	0.0383 (12)	−0.0032 (11)	0.0182 (10)	−0.0059 (10)
C3	0.0667 (15)	0.0382 (12)	0.0521 (14)	−0.0037 (10)	0.0228 (11)	−0.0026 (10)
C4	0.0457 (12)	0.0454 (12)	0.0444 (12)	0.0019 (10)	0.0190 (10)	0.0082 (10)
C5	0.0524 (13)	0.0518 (13)	0.0360 (11)	0.0026 (11)	0.0167 (10)	−0.0014 (10)
C6	0.0502 (12)	0.0402 (11)	0.0407 (12)	0.0004 (9)	0.0156 (10)	−0.0030 (10)
C7	0.0436 (12)	0.0493 (13)	0.0337 (11)	−0.0019 (10)	0.0108 (9)	−0.0021 (9)
C8	0.0395 (11)	0.0454 (12)	0.0303 (10)	0.0019 (9)	0.0096 (9)	0.0013 (9)
C9	0.0333 (10)	0.0472 (13)	0.0329 (10)	−0.0032 (9)	0.0084 (8)	0.0005 (9)
C10	0.0351 (11)	0.0488 (13)	0.0479 (13)	−0.0033 (9)	0.0120 (9)	0.0057 (10)
C11	0.0505 (14)	0.0609 (16)	0.0635 (16)	−0.0018 (11)	0.0129 (12)	0.0220 (13)
C12	0.0601 (15)	0.090 (2)	0.0450 (15)	−0.0131 (14)	0.0106 (12)	0.0225 (15)
C13	0.0604 (15)	0.089 (2)	0.0369 (13)	−0.0178 (14)	0.0182 (11)	−0.0050 (13)
C14	0.0478 (12)	0.0586 (14)	0.0399 (12)	−0.0076 (11)	0.0145 (10)	0.0018 (11)

### Geometric parameters (Å, °)

**Table:**

Cl1—C10	1.727 (2)	C4—C5	1.372 (3)
N1—C7	1.278 (3)	C5—C6	1.377 (3)
N1—N2	1.382 (2)	C5—H5	0.93
N2—C8	1.344 (3)	C6—H6	0.93
N2—H2A	0.895 (10)	C7—H7	0.93
N3—O2	1.214 (3)	C8—C9	1.495 (3)
N3—O1	1.215 (3)	C9—C10	1.388 (3)
N3—C4	1.468 (3)	C9—C14	1.410 (3)
O3—C8	1.226 (2)	C10—C11	1.388 (3)
C1—C6	1.389 (3)	C11—C12	1.378 (4)
C1—C2	1.390 (3)	C11—H11	0.93
C1—C7	1.454 (3)	C12—C13	1.365 (4)
C2—C3	1.374 (3)	C12—H12	0.93
C2—H2	0.93	C13—C14	1.372 (3)
C3—C4	1.373 (3)	C13—H13	0.93
C3—H3	0.93	C14—H14	0.93
			
C7—N1—N2	114.29 (16)	N1—C7—C1	121.13 (18)
C8—N2—N1	119.69 (15)	N1—C7—H7	119.4
C8—N2—H2A	123.1 (17)	C1—C7—H7	119.4
N1—N2—H2A	117.2 (17)	O3—C8—N2	124.00 (18)
O2—N3—O1	123.4 (2)	O3—C8—C9	123.06 (18)
O2—N3—C4	118.2 (2)	N2—C8—C9	112.86 (16)
O1—N3—C4	118.3 (2)	C10—C9—C14	118.30 (18)
C6—C1—C2	118.74 (19)	C10—C9—C8	122.97 (18)
C6—C1—C7	121.58 (19)	C14—C9—C8	118.69 (18)
C2—C1—C7	119.64 (19)	C11—C10—C9	120.3 (2)
C3—C2—C1	120.8 (2)	C11—C10—Cl1	117.86 (19)
C3—C2—H2	119.6	C9—C10—Cl1	121.83 (16)
C1—C2—H2	119.6	C12—C11—C10	119.7 (2)
C4—C3—C2	118.7 (2)	C12—C11—H11	120.1
C4—C3—H3	120.6	C10—C11—H11	120.1
C2—C3—H3	120.6	C13—C12—C11	121.0 (2)
C5—C4—C3	122.3 (2)	C13—C12—H12	119.5
C5—C4—N3	118.8 (2)	C11—C12—H12	119.5
C3—C4—N3	118.8 (2)	C12—C13—C14	119.9 (2)
C4—C5—C6	118.4 (2)	C12—C13—H13	120.1
C4—C5—H5	120.8	C14—C13—H13	120.1
C6—C5—H5	120.8	C13—C14—C9	120.7 (2)
C5—C6—C1	121.0 (2)	C13—C14—H14	119.6
C5—C6—H6	119.5	C9—C14—H14	119.6
C1—C6—H6	119.5		

### Hydrogen-bond geometry (Å, °)

**Table:**

*D*—H···*A*	*D*—H	H···*A*	*D*···*A*	*D*—H···*A*
N2—H2A···O3^i^	0.90 (1)	1.97 (1)	2.855 (2)	169 (2)

Symmetry codes: (i) *x*+1/2, −*y*+1/2, *z*+1/2.
